# Clinical presentation and surgical treatment of distal fibular non-union with talus chondral lesions in a pediatric patient: a case report

**DOI:** 10.1186/s12893-020-00782-z

**Published:** 2020-06-09

**Authors:** Marco Turati, Giulio Leone, Nicolò Zanchi, Robert J. Omeljaniuk, Lilia Brahim, Giovanni Zatti, Aurélien Courvoisier, Marco Bigoni

**Affiliations:** 1 Department of Paediatric Orthopedic Surgery, University Hospital Grenoble-Alpes, University Grenoble-Alpes, Grenoble, France; 2 Orthopedic Department, San Gerardo Hospital, University of Milano-Bicocca, Via Pergolesi 33, 20900 Monza, Italy; 3 Department of Medicine and Surgery, University of Milano-Bicocca, 20900 Monza, Italy; 4 Department of Biology, Lakehead University, Thunder Bay, ON P7B5E1 Canada

**Keywords:** Children, Nonunion, Fibula, Malleolus fracture

## Abstract

**Background:**

In children, fracture non-union is uncommon yet, curiously, non-union of distal fibula fractures are rarely reported. Historically, the most common treatment of a lateral malleolus fracture after an ankle sprain is conservative, which usually leads to fracture union. However, even in clinically stable ankles, subsequent pain arising from fracture site could suggest non-union, thereby necessitating reexamination and possible secondary treatment.

**Case presentation:**

We report the case of an 8-year-old girl with an epiphyseal distal fibula fracture complicated with a symptomatic non-union associated with the chondral flap of the talar dome after conservative treatment. Surgical excision of the fragment and chondroplasty was performed and resulted in an excellent clinical outcome.

**Conclusion:**

This case report illustrates the necessity of particularly meticulous evaluation of pediatric post-traumatic ankle pain. Surgical treatment as well as talar chondral evaluation should be taken into consideration in the treatment of pediatric distal fibular nonunion.

## Background

In the pediatric population, 15% of all the fractures involve the physis in different ways. Of these fractures, between 3 and 12% are distal fibula fractures [1] which are most commonly associated with ankle sprains, the majority of which are sports-related, and which represent than one-third of children’s sporting injuries [1]. These traumas associated with distal fibula fracture are accompanied by a very low incidence of long-bone non-union (0 to 1.7%) [2–5]. However, the incidence of non-union increases with bone infections and with premature, and repeated manipulation [4, 6, 7].

Fortunately, the occurrence of non-union consequent to a pediatric fibula fracture is rare in the population [2, 6, 8] but, is significant to the affected individual. This report presents the case of a distal fibula non-union in an 8-year-old girl who was initially conservatively treated but who then required surgical remediation. Additionally, to our knowledge this is the first report of a chondral talar lesion associated with a distal fibula non-union.

## Case presentation

**Fig. 1:**
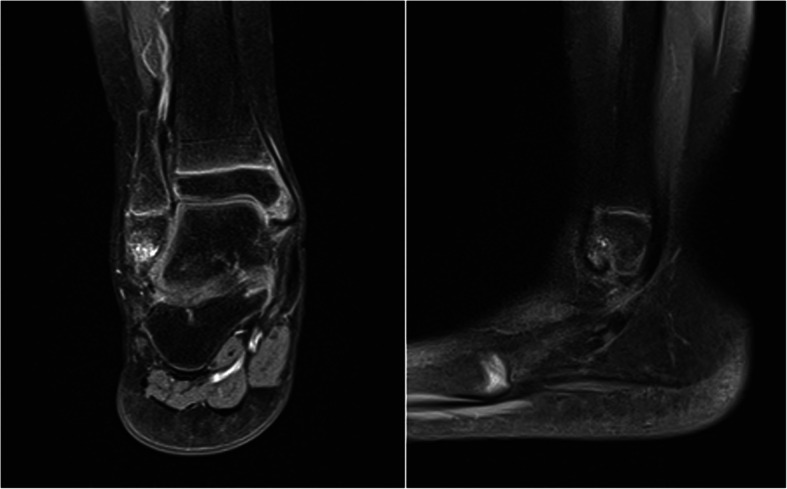
Anteroposterior and lateral MRI. The non united fragment is visible at the apex of the me the lateral malleolus, with an anomal signal and a signal suggestive of bone marrow edema on T2 images

An 8-year-old girl presented with right ankle pain during routine daily activity. Nine months before presentation she suffered a major ankle sprain, at which point X-ray imaging revealed no evidence of any fractures; consequently, the leg was immobilized in a cast for 14 days followed by a gradual return to daily routine activities. Nonetheless, the patient continued to experience persistent pain and reduced her participation in sports. The patient, otherwise, had no other significant previous medical or surgical history. At the time of presentation, the ankle was stable, but accompanied by pain at the tip of the right lateral malleolus. An MRI was then performed (Fig. 1) and revealed a single alteration at the apex of the fibula, as well as the delayed union of a 1 cm fragment at the tip of the lateral malleolus. In an effort to promote union non-invasively, conservative treatment was repeated, postulating that the initial return to daily activities was premature. The leg was immobilized in a cast for 35 days, followed by physiotherapy.

**Fig. 2:**
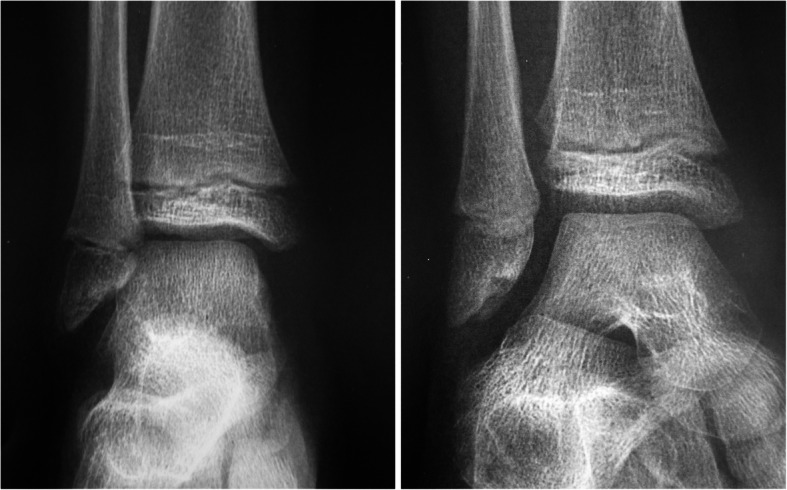
Anteroposterior and oblique x-rays prior to surgery. The non united fragment is visible at the apex of the lateral malleolus. The rest of the ankle joint is normal

**Fig. 3:**
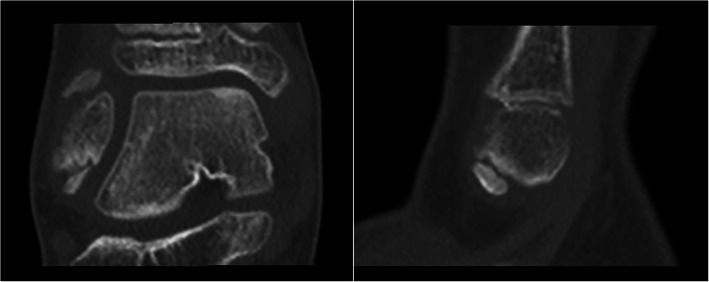
Anteroposterior and lateral CT scan. The non united fragment is visible and classified as a type 7b in the Ogden classification

An x-ray of the ankle (Fig. 2) performed 30 days after cast removal, and during the physiotherapy program, revealed the persistence of the non-healed fracture and the non-united fragment at the tip of the fibula. MRI and CT scan imaging (Fig. 3), confirmed the persistence of the fracture and the absence of other pediatric pathologies (as osteochondrosis) and anatomic variances (as os subfibulare); this diagnosis was also confirmed by a pediatric musculoskeletal radiologist. In consideration of the child’s youth (aged 8 years) conservative treatment was repeated with a leg cast for 35 days.

Subsequent x-ray imaging (4 months after initial presentation) showed a non-healed fracture resulting in the final diagnosis of non-union; consequently, surgery was elected as the preferred treatment.

**Fig. 4:**
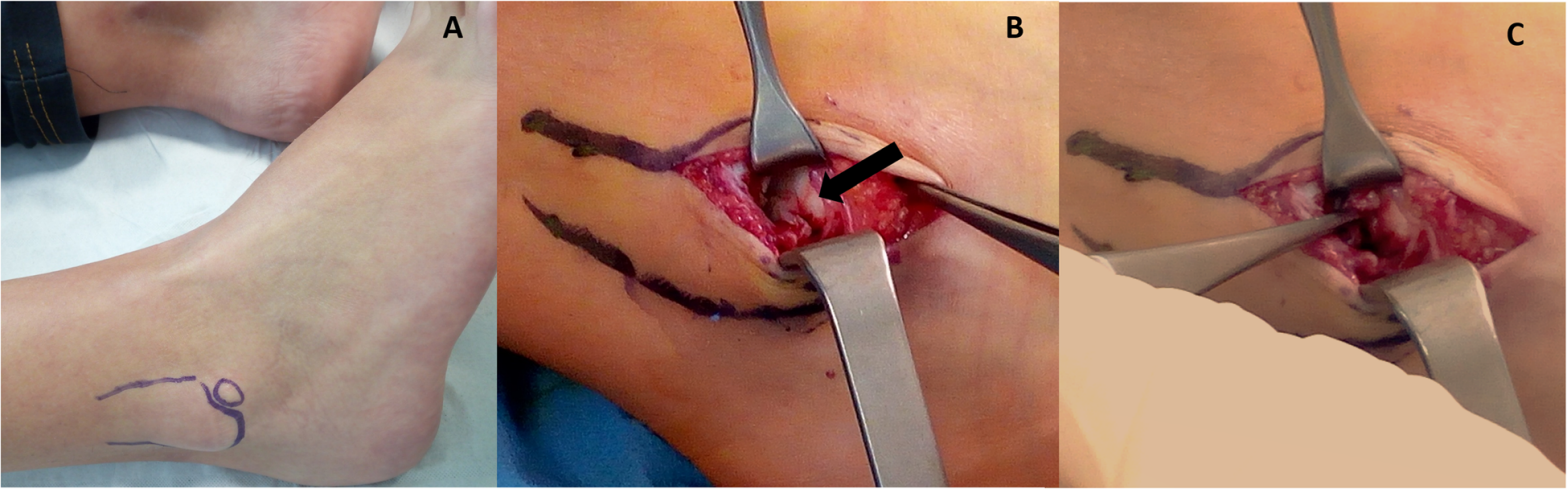
Intraoperatory pictures. The fragment is evidenced before surgery (**a**); after surgical approach avulsion of the distal fragment was performed and the talofibular joint was exposed (**b**-**c**): an unstable chondral flap of the lateral wall of the talar dome was found (arrow in figure **b**).

The patient was placed supine on the operating table and a direct anterolateral approach to the distal fibula was performed after fluoroscopic localization of the distal fragment (Fig. 4-a). Intraoperatively, the distal fragment was identified. The fragment was clearly mobile with ankle stress examination tests. Fibers of the anterior talofibular ligament were attached to the footprint on the avulsed fragment. Debridement of the non-union interface was performed. Considering the dimension of the distal fibular fragment, an ORIF was excluded. After anterior talofibular ligament fibers detachment, an avulsion of the fragment was performed. The talofibular joint was exposed. Interestingly, an unstable chondral flap of the lateral wall of the talar dome was found (Fig. 4-b). The position of this chondral flap during dynamic examination suggested a mechanical impingement between the talus and the avulsed distal fragment as the etiological cause. A debridement of this chondral flap was performed. The detached portion of the anterior talofibular ligament was reinserted with transosseous suture to the distal fibula.

The patient was treated with cast immobilization for 6 weeks; thereafter, physical therapy focused on range of motion, proprioceptive rehabilitation and gradual return to normal ankle function for a further 3 months. At 3 months after surgery, the patient was without any symptoms, with a full ankle range of motion and without any indication of instability. A progressive return to sports-related activity was allowed. No re-occurrence was reported at the 2-year follow-up examination.

## Discussion and conclusions

The physeal fracture type in the case now presented corresponds to a Type 7b under Ogden’s growth physis classification system, an extension of that by Salter and Harris. This Type 7b fracture is completely intra-epiphyseal and represents the propagation of the fracture from the articular cartilage to the secondary ossification center without involving it [7].

Clinical assessment confirmed that ankle pain was the main problem with no involvement of the lateral ligamentous complex in terms of stability. Ankle stability was meticulously assessed as its preservation (which depends on many anatomical structures including: (i) lateral ankle ligaments, (ii) distal tibio-fibular joints, (iii) muscles, (iv) tendons, and (v) innervation) [9] must figure pre-eminently in the selection of operative treatment [2]. Pediatric ankle pain is common but a meticulous clinical evaluation is essential because different etiologies such as painful accessory bone, osteochondrosis and tarsal coalition should be excluded [10–14]. In cases involving the youngest skeletally immature patients with traumatic injuries, imaging techniques, such as MRI and CAT scans are essentially necessary to visualize chondral structures, not resolvable by x-rays, but which also may be damaged [14–16]. In this case, x-rays performed after the ankle sprain were negative as the tip of the lateral malleolus was not ossified at 8 years old and avulsion fractures are very difficult to visualize in the youngest patients. In cases of persistent pain, following initial examination and treatment, these very young pediatric ankle injuries should be clinically re-assessed and accompanied by MRI soon thereafter in order to assess the conditions of poorly ossified bone and cartilaginous tissue.

The origin of the pain was the fracture nonunion with interposition of fibrous tissue between the bones fragment as confirmed intraoperatively. Considering the small size of the fragment (1 cm approximately) and the ankle stability, and as ORIF was not possible, the fragment was excised which completely mitigated all pain.

There are few reports of pediatric distal fibula delayed unions and non-unions [2, 6, 8]. Mirmiran et al. reported the case of a 12-year-old boy, with a personal history of ankle sprains which were always treated conservatively, that suffered from a distal fibula non-union after an inversion injury of the ankle. They performed ORIF with 2 cancellous screws and a bioabsorbable screw for the syndesmosis however, they noted its insufficiency with stress maneuvers during surgery. Outcome results were good with a return to sports and no further ankle sprains [2]. Haramati et al. reported 3 cases of distal fibula fracture non-unions; of these, two did not involve the physis and were treated with excision. In the third one, the fracture extended to the growth physis and was treated with ORIF. In all three cases outcomes were good with return to normal activities [6]. El Ashry et al. reported 2 cases of ankle instability associated with distal fibula fracture non-union, which were both treated with ORIF [17]. Mandalia et al. reported the case of an 11-year-old boy with a type 7 fracture of the distal fibula that healed with conservative treatment [18].

In the present case an unstable chondral flap of the talar dome was found subsequent to distal fragment avulsion. During intraoperative dynamic evaluation, this chondral flap was found to contribute to a mechanical impingement between the avulsed distal fragment and the talus. The inappropriate anatomical position of the fragment was likely the principle cause of the patient’s persistent pain [19]. Moreover, the chondral lesion required surgical treatment in order to reduce the mechanical impingement and to treat any chondral associated injuries.

Archetypal conservative treatment for distal fibula fractures, involves leg immobilization in a cast followed by physiotherapy, typically yields good outcomes [18, 20]. Nonetheless, those studies consistently identify the real potential for non-union and implied the need for appropriate vigilance. Initially, adequate time must be allocated for spontaneous fracture healing; thereafter, any cases of delayed union must be closely followed to determine if healing eventually completes, or, a non-union occurs. Persistent pain subsequent to distal fibula fracture is not exclusively due to non-union; consequently, a meticulous examination should be performed, [10–13], and should include MRI and CT scan imaging in order to establish a correct diagnosis and in order to explicitly confirm any necessity of surgical treatment [12–14, 17].

Os subfibulare is an anatomic variant considered either as a failure of an accessory ossification center to unite to the distal fibula, or, a supernumerary bone at the apex of the fibula [21, 22].

In 1990 Ogden and Lee reported a distal focus of epiphyseal ossification occurring at the lateral and medial malleoli [23] which were considered to be normal variants. Nonetheless, os subfibulare may be asymptomatic and, as a consequence, are only incidentally reported. However, symptomatic os subfibulare should be meticulously evaluated and a traumatic diagnosis should be excluded. Indeed, intraoperative findings in adults suspected of symptomatic os subfibulare revealed non-unions of an avulsion fracture of the anterior talofibular ligament [24].

For this reason, some authors suggest that os subfibulare represents an avulsion fracture of the anterior talofibular ligament or of the calcaneofibular ligament [15, 16, 21]. Pill et al. reported 23 patients with a chronic symptomatic os subfibulare in children, observed after an ankle sprain injury; after unsuccessful conservative treatment, the fragment was excised, with reconstruction of the anterior talofibular ligament. All patients except one returned to preinjury recreational levels [25]. Os subfibulare must always be considered when we find a bone fragment distal to the apex of the fibula.

Clinical history and previous X-ray images are helpful for a correct diagnosis. It is also useful to consider the position and the shape of the fragment in order to differentiate a non-union from an accessory bone. In the present case, clinical traumatic history, the particular shape and borders of the fragments, its position and CT and MRI imaging suggested that the pain originated by the non-union of an avulsion fracture. If the fragment is too small for ORIF, despite its origin, excision and ligamentous reconstruction is a valid surgical treatment option.

The literature suggests two different surgical approaches. For large fragments with complex ligamentous involvement, ORIF must be considered. Cancellous screws can be used for large fragments. By contrast, the use of anchors is suggested for small fragments [7]. It is essential to remove all fibrous tissue in the fracture line prior to fixation in order to facilitate bone healing. If, however, the fragment is too small for ORIF, excision could be the best choice, but particular attention is required to avoid compromising the ligamentous apparatus as this can result in ankle instability. In either approach, whether conservative or surgical treatment, clinical examination and x-rays must be performed in order to document fracture healing and resolution of symptoms.

To the best of our knowledge there is, as yet, no report in the literature on pediatric distal fibular non-union and associated chondral tears. The present case highlights the importance of recognizing the significance of persistent ankle pain after a sprain in pediatric patients. Both accurate and very precise clinical examination as well as advanced diagnostic imaging are needed in order to diagnose any possible complications. Considering distal fibular non-union, anatomical reduction of fibular fractures is another important aspect, suggesting that mechanical impingement between the distal fibula and talus may not only contribute to this painful condition but also to chondral articular damage. Further bio-mechanical and long-term studies are required in order to better understand this rare but probably underestimated condition.

## Data Availability

The datasets generated during and/or analysed during the current study are available from the corresponding author on reasonable request.

## Abbreviations

MRI: Magnetic Resonance Imaging
CT: Computed Tomography
ORIF: Open Reduction and Internal Fixation

## References

[1] Swenson DM, Yard EE, Fields SK, Comstock RD (2009). Patterns of Recurrent Injuries among US High School Athletes, 2005–2008. Am J Sports Med.

[2] Mirmiran R, Schuberth J (2006). Non Union of an Epiphyseal Fibular Fracture in a Pediatric Patient. J Foot Ankle Surg.

[3] Jones BG, Duncan RDD (2003). Open tibial fractures in children under 13 years of age—10 years experience. Injury.

[4] Mirdad T (2000). Operative treatment of femoral shaft fractures in children: a nine-year experience in a Saudi Arabian population. Injury..

[5] Eren OT, Kucukkaya M, Kockesen C, Kabukcuoglu Y, Kuzgun U (2003). Open reduction and plate fixation of femoral shaft fractures in children aged 4 to 10. J Pediatr Orthop.

[6] Haramati N, Roye DP, Adler PA (1994). C Ruzal-Shapiro, Non-union of pediatric fibula fractures: Easy to overlook, painful to ignore. Pediatr Radiol.

[7] Ogden JA (1981). Injury to the growth mechanisms of the immature skeleton. Skeletal Radiol..

[8] Lewallen RP, Peterson HA (1985). Nonunion of long bone fractures in children: a review of 30 cases. J Pediatr Orthop.

[9] Hertel J (2002). Functional Anatomy, Pathomechanics, and Pathophysiology of Lateral Ankle Instability. J Athl Train.

[10] Turati M, Glard Y, Afonso D, Griffet J (2017). M Bigoni, Osteochondral alteration in a child treated with levetiracetam: a rare case of juvenile osteochondritis dissecans of the talar head. J Pediatr Orthopaedics B.

[11] Turati M, Afonso D, Salazard B, Declerck MM, Bigoni M (2015). Y Glard, Bilateral osteochondrosis of the distal tibial epiphysis: a case report. J Pediatr Orthopaedics B.

[12] Alfred Atanda J, Shah SA, O’Brien K (2011). Osteochondrosis: Common Causes of Pain in Growing Bones. AFP.

[13] Docquier P-L, Maldaque P (2019). M Bouchard, Tarsal coalition in paediatric patients. Orthop Traumatol Surg Res.

[14] The difficult diagnosis of cartilaginous tibial eminence fractures in young children. - PubMed - NCBI. https://www.ncbi.nlm.nih.gov/pubmed/23636131 (consultato apr. 22, 2020). 10.1007/s00167-013-2518-8 23636131

[15] Andreacchio A, Marengo L (2018). F Canavese, Solitary osteochondroma of the sinus tarsi. J Pediatr Orthop B.

[16] Turati M, Glard Y, Griffet J, Afonso D, Courvoisier A, Bigoni M (2017). Osteochondrosis of the medial malleolar epiphysis: A case report and review of the literature. Int J Surg Case Rep.

[17] El Ashry SR, El Gamal TA (2017). SR Platt, Atypical Chronic Ankle Instability in a Pediatric Population Secondary to Distal Fibula Avulsion Fracture Nonunion. J Foot Ankle Surg.

[18] Mandalia V, Shivshanker V (2005). Accessory ossicle or intraepiphyseal fracture of lateral malleolus: are we familiar with these?. Emerg Med J.

[19] Turati M, Bigoni M, Omeljaniuk R, Griffet J, Zatti G, Courvoisier A. Pediatric navicular dorsal osteochondroma: a rare case of navicular–cuneiform impingement. J Pediatr Orthopaedics B, Publish Ahead of Print. 2019. 10.1097/BPB.0000000000000625. 10.1097/BPB.0000000000000625 30855546

[20] Zura R (2018). Risk factors for nonunion of bone fracture in pediatric patients. Medicine (Baltimore).

[21] O’Rahilly R (1953). A survey of carpal and tarsal anomalies. J Bone Joint Surg.

[22] Shands AR, Wentz IJ (1953). Congenital anomalies, accessory bones, and osteochondritis in the feet of 850 children. Surg Clin North Am.

[23] Ogden JA, Lee J (1990). Accessory ossification patterns and injuries of the malleoli. J Pediatr Orthop.

[24] Berg EE (1991). The symptomatic os subfibulare. Avulsion fracture of the fibula associated with recurrent instability of the ankle. J Bone Joint Surg Am.

[25] Pill SG, Hatch M, Linton JM, Davidson RS (2013). Chronic symptomatic os subfibulare in children. J Bone Joint Surg Am.

